# Linezolid and Rifampicin Combination to Combat *cfr*-Positive Multidrug-Resistant MRSA in Murine Models of Bacteremia and Skin and Skin Structure Infection

**DOI:** 10.3389/fmicb.2019.03080

**Published:** 2020-01-14

**Authors:** Yu-Feng Zhou, Liang Li, Meng-Ting Tao, Jian Sun, Xiao-Ping Liao, Ya-Hong Liu, Yan Q. Xiong

**Affiliations:** ^1^National Risk Assessment Laboratory for Antimicrobial Resistance of Animal Original Bacteria, College of Veterinary Medicine, South China Agricultural University, Guangzhou, China; ^2^Guangdong Provincial Key Laboratory of Veterinary Pharmaceutics Development and Safety Evaluation, South China Agricultural University, Guangzhou, China; ^3^The Lundquist Institute for Biomedical Innovation at Harbor-UCLA Medical Center, Torrance, CA, United States; ^4^Jiangsu Co-Innovation Center for the Prevention and Control of Important Animal Infectious Diseases and Zoonoses, Yangzhou University, Yangzhou, China; ^5^David Geffen School of Medicine at UCLA, Los Angeles, CA, United States

**Keywords:** MRSA, *cfr*, phenotype, biofilm, bacteremia, skin and skin structure infection, combination therapy

## Abstract

Linezolid resistance mediated by the *cfr* gene in MRSA represents a global concern. We investigated relevant phenotype differences between *cfr*-positive and -negative MRSA that contribute to pathogenesis, and the efficacy of linezolid-based combination therapies in murine models of bacteremia and skin and skin structure infection (SSSI). As a group, *cfr*-positive MRSA exhibited significantly reduced susceptibilities to the host defense peptides tPMPs, human neutrophil peptide-1 (hNP-1), and cathelicidin LL-37 (*P* < 0.01). In addition, increased binding to fibronectin (FN) and endothelial cells paralleled robust biofilm formation in *cfr*-positive vs. -negative MRSA. *In vitro* phenotypes of *cfr*-positive MRSA translated into poor outcomes of linezolid monotherapy *in vivo* in murine bacteremia and SSSI models. Importantly, rifampicin showed synergistic activity as a combinatorial partner with linezolid, and the EC_50_ of linezolid decreased 6-fold in the presence of rifampicin. Furthermore, this combination therapy displayed efficacy against *cfr*-positive MRSA at clinically relevant doses. Altogether, these data suggest that the use of linezolid in combination with rifampicin poses a viable therapeutic alternative for bacteremia and SSSI caused by *cfr*-positive multidrug resistant MRSA.

## Introduction

MRSA is particularly challenging due to its inherent pathogenicity and multidrug resistant phenotypes contributing to a variety of infectious diseases, ranging from skin and skin structure infection (SSSI) to bacteremia (Tong et al., 2015; Wang et al., 2019). An increased global incidence of MRSA infections associated with high mortality has been observed over the past decades (Bassetti et al., 2014; Hassoun et al., 2017). For example, in the United States, *S. aureus* is most often contracted as a nosocomial infection leading to more than 80,000 illnesses and 11,000 deaths yearly (Lepak and Andes, 2016). Therefore, new alternative strategies for the treatment of such infections are urgently needed.

Linezolid has become an important drug for treating nosocomial infections due to MRSA, including those with reduced vancomycin susceptibility (e.g., VISA) (Dryden, 2011). However, linezolid resistance due to acquisition of the *cfr* (chloramphenicol and florfenicol resistance) gene has compromised MRSA treatment options (Long et al., 2006). The *cfr* gene encodes a 23S rRNA methyltransferase that confers combined resistance to phenicols, lincosamides, oxazolidinones, pleuromutilins, and streptogramin A (PhLOPS_A_ phenotype) (Long et al., 2006; Witte and Cuny, 2011). In addition, there is only a low fitness cost to the host for *cfr* carriage and this facilitates its spread (LaMarre et al., 2011). Infections due to *cfr*-positive MRSA are increasing and pose a serious threat to the clinical success of oxazolidinone antibiotics (Witte and Cuny, 2011). Although the level of resistance to linezolid conferred by *cfr* is moderate, the ability of *cfr* to enhance bacterial survival in the presence of linezolid has been shown *in vivo* in a murine pneumonia model (Zhou et al., 2018). Linezolid-resistant MRSA strains carrying *cfr* were also associated with prolonged use of linezolid in patients (Endimiani et al., 2011). These data suggest that in addition to the *cfr*-mediated linezolid resistance, *cfr*-positive MRSA may possess phenotypes associated with pathogenesis that contribute to poor *in vivo* treatment outcomes.

In this study, we profiled relevant phenotype differences between *cfr*-positive and -negative MRSA that contribute to bacteremia and SSSI. We examined whether these MRSA were susceptible to host defense cationic peptides (HDP) and assayed their biofilm forming abilities and binding to fibronectin (FN) and endothelial cells. In addition, we correlated *in vitro* phenotypes to linezolid resistance *in vivo* in murine SSSI and bacteremia models to characterize *cfr*-positive and -negative MRSA.

## Materials and Methods

### Bacterial Strains and Background Information

Ten well-characterized MRSA strains were used in this study (Li et al., 2018; Zhou et al., 2018). Human clinical MRSA strains (161402, 161400, 161494, and 161813) were kindly provided by the Third Affiliated Hospital of Sun Yat-sen University (Guangzhou, China) that obtained from hospitalized patients with pulmonary infections. MRSA strains of animal origin (N50, 6Y2C, HYP6, N4-2, HYXC4, and 2B3) were collected from blood and abscess cultures of sick animals at the Animal Diagnostic Laboratory of South China Agricultural University. All strains were identified by MALDI-TOF MS system (Ostergaard et al., 2015). Four MRSA strains of human origin were typed as ST 764 and *spa*-type t1081, and the remaining six strains were typed as ST 398 and ST 9 (Li et al., 2018; Zhou et al., 2018). The ST 764 MRSA has emerged as a novel hybrid variant of the ST 5 HA-MRSA lineage with the characteristics of CA-MRSA in Asia, causing invasive infections (necrotizing fasciitis and bacteremia) in both hospital and community settings (Takano et al., 2013). The ST 398 MRSA has been reported in China and Europe that was responsible for zoonotic infections in patients with pneumonia and SSSIs (Stegger et al., 2010; van der Mee-Marquet et al., 2011).

### Linezolid-Based Combination Susceptibility Testing and Time-Kill Curves

The MICs of linezolid and other ten antibiotics (oxacillin, cefotaxime, amikacin, azithromycin, tetracycline, vancomycin, clindamycin, retapamulin, ciprofloxacin, and rifampicin) against clinical MRSA isolates were conducted by the broth microdilution method as recommended (CLSI, 2015). *S. aureus* ATCC 29213 served as the quality control strain. Fold reduction in MIC was determined by dividing the MIC of the antibiotic alone by its MIC in the presence of 0.5 mg/L linezolid. Three biological replicates were done for each combination and the means of fold reduction were used for generating heat maps. *In vitro* interactions between linezolid and rifampicin were evaluated by the checkerboard method, and a fractional inhibitory concentration index (FICI) of ≤0.5 was deemed synergistic (Zhou et al., 2018).

*In vitro* time-kill curves were performed to compare the activity of linezolid and rifampicin alone and in combination against two representative *cfr*-positive and -negative strain sets. In brief, a starting inoculum of ∼10^6^ cfu/mL logarithmic phase MRSA cells was used to expose to linezolid (16 mg/L) with or without rifampicin (0.5 mg/L). The drug concentrations were chosen to mimic the free serum steady-state peak concentrations (*f*C_max_) at the usual clinical doses in human (i.e., 600 mg for linezolid, 300 mg for rifampicin) (Andes et al., 2002; Sirgel et al., 2005; Chik et al., 2010; Dryden, 2011). MRSA densities were determined by the serial viable counts collected over 24 h incubation and expressed as log_10_ cfu/mL. Synergistic effect was defined as the combination caused ≥2 log_10_ cfu/mL reduction vs. the single drug.

### *In vitro* Concentration-Effect Relationship

Concentration-effect curves were used to evaluate linezolid potency against *cfr*-positive and -negative MRSA. Briefly, an overnight culture of MRSA cell was washed, adjusted to 0.5 McFarland units and diluted in cation-adjusted Mueller Hinton broth to a final density of 10^6^ cfu/mL. The testing procedure consisted of two groups, and each group included tubes with two-fold increasing concentrations of linezolid from 0.015 to 32 mg/L, in the presence and absence of 0.5 × MIC rifampicin. After 16 h of incubation at 37°C, absorbance of each tube was measured at OD_600__nm_ to quantify bacterial growth and normalized with the no drug control. The relationship between linezolid concentrations and antibacterial potency was calculated using the Hill sigmoid E_max_ equation: E = E_max_ + (E_0_ – E_max_)/1 + 10^∧^[(log EC_50_ – C) × Hill slope] using GraphPad Prism 8 software (Zhou et al., 2017).

### *In vitro* HDP Susceptibility

The tPMPs were prepared from thrombin-stimulated platelets isolated from fresh rabbit blood and their bioactivity was quantified using *Bacillus subtilis* ATCC 6633 as previously described (Yeaman et al., 1992). Human neutrophil peptide-1 (hNP-1) and cathelicidin LL-37 were purchased from Peptides International (Louisville, KY, United States) and Eurogentec (Fremont, CA, United States), respectively. *In vitro* HDP susceptibilities were assessed by adding tPMP (2 mg/L equivalent) to 10^3^ cfu/mL MRSA cells and hNP-1 (5 mg/L) or LL-37 (20 mg/L) to 10^5^ cfu/mL MRSA cells (Xiong et al., 2009; Seidl et al., 2011a). The HDP concentrations were selected to cover the peptide concentrations that did not rapidly kill MRSA cells over 2 h of incubation based on previous studies (Seidl et al., 2011a). Results were expressed as the percentage of the initial inoculum that survived exposure to HDPs.

### Adherence to Fibronectin and Endothelial Cells

Six-well tissue culture plates were coated using 50 mg/L purified human FN (Sigma Chemical, St. Louis, MO, United States) overnight at 4°C, and then treated with 3% bovine serum albumin for 3 h to prevent non-specific adhesion (Xiong et al., 2009). The human microvascular endothelial cell line (HMEC-1) was cultured as previously described (Seidl et al., 2012). Logarithmic-phase MRSA cells were added to FN-coated plates (5 × 10^3^ cfu/mL) and endothelial cell monolayer-coated plates (5 × 10^5^ cfu/mL; MOI = 1:1), and then incubated for 1 h at 37°C under static conditions. For FN binding assay, unbound bacteria were removed by washing the plates with PBS, and melted tryptic soy agar (TSA; 2 mL) was added into each well and allowed to solidify. For endothelial cell binding assay, unbound bacteria were removed by washing the plates with Hanks balanced salt solution (HBSS) and permeabilized using 1.0% Triton X-100 (Seidl et al., 2012), after which bacterial numbers per well were determined by serial dilutions and plating on TSA. Adherence was expressed as the percentage of the initial inoculum bound.

### Biofilm Formation, Extracellular Polysaccharide (EPS) and DNA Determinations

The ability of MRSA to form biofilm was determined as described previously (Seidl et al., 2011a). Briefly, overnight cultured MRSA at 0.5 McFarland units (∼10^8^ cfu/mL) was diluted 1:100 into brain heart infusion (BHI) broth supplemented with 0.5% glucose. 200 μL of the suspension was transferred into 96-well plates and incubated for 18 h at 37°C. After incubation, the plates were washed with PBS, air dried and stained with 0.1% safranin. The adhering dye was dissolved in 30% acetic acid, and absorption was measured at OD_490__nm_ to quantify biofilm formation.

The water-soluble and -insoluble EPS synthesized by the biofilms was examined using the anthrone-sulfuric method (Chen et al., 2016). Briefly, 24 h biofilms were rinsed, removed and dispersed by sonication at 20 kHz for 5 s. The suspension was centrifuged at 6000 × *g* for 10 min at 4°C, and the supernatant was collected for water-soluble EPS determinations. The pellets were resuspended in PBS, washed and air-dried to ensure all the water-soluble EPS was discarded. The dry weight of each biofilm was measured to adjust biomass differences between *cfr*-positive and -negative MRSA. The water-insoluble EPS was extracted using 1.0 M NaOH under agitation for 2 h at 37°C and quantified using an anthrone-sulfuric acid colorimetric assay (Chen et al., 2016).

Release of extracellular DNA (eDNA) was determined from 18 h MRSA biofilm using a microplate fluorescence assay with Hoechst dye 33258 (Leggate et al., 2006). Protocols for extraction and purification of eDNA from MRSA biofilms were described in detail elsewhere (Rice et al., 2007). The eDNA was quantified using an EnSight fluorescence plate reader at Ex_350_/Em_460_ (PerkinElmer, Waltham, MA, United States). Purified salmon sperm DNA was used to generate a standard curve. To account for differences in biomass, the average OD_490__nm_ of each unwashed biofilm was determined to calculate the amount of eDNA per relative biomass.

### Hemolytic Activity and Nuclease Production

Hemolytic activity was evaluated by spotting 2 μL of MRSA suspension (∼10^8^ cfu/mL) onto 5% sheep blood agar plates and incubated at 37°C for 24 h (Seidl et al., 2011b). The diameters of the zones of clearance (cm) indicating hemolytic activity were measured. Nuclease production was assessed by spotting 15 μL of filtered culture supernatants of the strains into wells cut into DNase test agar (Beenken et al., 2010). Plates were incubated overnight at 37°C. Nuclease activity was then assessed by overlaying the agar with 1 M HCl to precipitate undigested DNA and define the zone diameters (cm) of clearance (Beenken et al., 2010).

### *In vivo* Murine Bacteremia and SSSI Models

Six-week-old, pathogen-free female ICR mice (25–27 g from Guangdong Medical Lab Animal Center, Guangzhou, China) were used in this study. All animal experimental procedures were approved by the South China Agricultural University (SCAU) Institutional Ethics Committee (2017B075 and 2017018) and performed in accordance with the SCAU Institutional Laboratory Animal Care and Use guidelines. For bacteremia model, mice were infected *via* the tail vein with a 0.5 mL bacterial suspension delivering ∼10^5.5–6.0^ cfu/mouse (Thakker et al., 1998). For SSSI model, 0.1 mL of bacterial suspension consisting of ∼10^7.0^ cfu was inoculated subcutaneously into the flanks of mice (Tseng et al., 2011). Four representative MRSA strains were selected for *in vivo* studies based on their *in vitro* phenotypes and MLST types that included *cfr*-positive and -negative MRSA.

To assess the therapeutic efficacy of linezolid and rifampicin alone and in combination, mice were randomized at 24 h (bacteremia model) and 48 h (SSSI model) post-infection to receive: (i) no therapy (control); (ii) linezolid at 100 mg/kg, orally twice daily; (iii) rifampicin at 5 mg/kg, orally twice daily; or (iv) a combination of linezolid and rifampicin. The linezolid and rifampicin doses were selected to mimic the pharmacokinetic profiles of recommended human clinical doses (i.e., 600 mg and 300 mg, orally twice daily for linezolid and rifampicin, respectively) (Chik et al., 2010; Zhou et al., 2018). Treatments lasted for 3 and 5 days for the bacteremia and SSSI models, respectively. Groups of five or six mice were included at each dose regimen. Control and antibiotic-treated mice were sacrificed either at the beginning of treatment (untreated controls) or 12 h after the last antibiotic dose, respectively. At sacrifice, the target tissues (blood, spleen and kidney for bacteremia model, and skin abscess for SSSI model) were removed and quantitatively cultured. Bacterial densities in infected tissues were calculated as the mean log_10_ cfu/g. of tissue and log_10_ cfu/mL of blood (± SD). In addition, the mean areas of superficial skin lesions were quantitated for statistical comparisons in the SSSI model.

### Statistical Analysis

*In vitro* studies were performed with three biological replicates in triplicate. Two-tailed Student’s *t*-test was used to compare relevant phenotype differences between *cfr*-positive and -negative MRSA groups. Mann-Whitney non-parametric test was used to analyze MRSA densities in target tissue among different groups.

## Results

### Linezolid-Based Combination Potentiated Activity Against *cfr*-Positive MRSA

**FIGURE 1:**
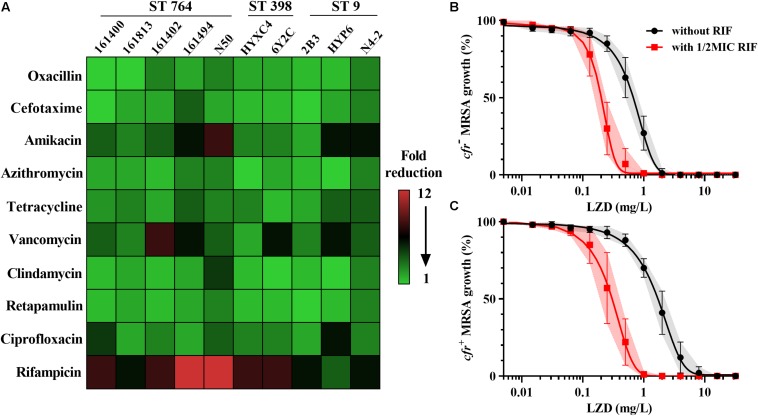
Linezolid-based combination susceptibility testing and potency analysis. **(A)** Heat map showing the mean fold reduction of MIC in the presence of 0.5 mg/L linezolid for MRSA strains. The strains carrying the *cfr* gene are underlined; Concentration-effect analysis of linezolid against *cfr*-negative **(B)** and *cfr*-positive **(C)** MRSA strains in the presence (red) and absence (black) of 0.5 × MIC rifampicin. Data were shown as mean with 95% CIs from three biological replicates.

As expected, all the study MRSA isolates were resistant to oxacillin with MICs ranging from 8 to 128 mg/L. The MICs of linezolid were markedly higher in MRSA isolates harboring the *cfr* gene (1–8 mg/L) than in those lacking the *cfr* (0.5–2 mg/L; Table 1). Fold reductions in MICs of amikacin and vancomycin were observed for part of *cfr*-positive MRSA isolates when combined with the sub-MIC levels of linezolid at 0.5 mg/L. However, the broad-spectrum antibiotics including cefotaxime and ciprofloxacin displayed a limited MIC reduction. Notably, in the presence of linezolid, rifampicin achieved the highest therapeutic potential as a combinatorial partner with a greater than 8-fold reduction in MIC against 8/10 MRSA isolates, and this was independent of *cfr* expression (Figure 1A). The combination of linezolid and rifampicin resulted in synergistic activity against 5/6 *cfr*-positive MRSA isolates and 2/4 *cfr*-negative MRSA isolates, with FICIs ranging from 0.375 to 0.5 (Table 1).

**TABLE 1:** Genotypic summary and MICs for study MRSA isolates.

**MRSA strains**	**MLST**	***spa* types**	**MIC (mg/L)^a^**			**FICI^b^**
			**OXA**	**LZD**	**RIF**	
***cfr*-positive**						
161402	ST764	t1084	128	8	1	0.375
161494	ST764	t1084	128	4	1	0.5
N50	ST764	t899	32	2	8	0.25
6Y2C	ST398	t7829	16	2	8	0.5
HYP6	ST9	t899	64	1	0.12	0.5
N4-2	ST9	t899	32	1	16	0.75
***cfr*-negative**						
161400	ST764	t1084	32	1	0.5	0.5
161813	ST764	t1084	32	0.5	1	0.75
HYXC4	ST398	t7880	8	2	4	0.375
2B3	ST9	t899	64	1	0.25	0.625
**ATCC strain**						
29213	ST5	t002	0.25	1	0.008	0.5

**^*a*^*OXA, oxacillin; LZD, linezolid; RIF, rifampicin. *^*b*^*Interpreted as synergy (FICI ≤ 0.5), no interaction (0.5 < FICI ≤ 4) or antagonism (FICI > 4).*

Control cultures increased ∼3-log_10_ cfu/mL for both *cfr*-positive and -negative MRSA over a 24 h of incubation. Rifampicin alone had the similar bacterial growths vs. their control groups (Supplementary Figure S1). Of note, despite having the same MICs, linezolid alone at 16 mg/L resulted in greater bacterial killing for *cfr*-negative strain HYXC4 (1.70-log_10_ cfu/mL) vs. the *cfr*-positive strain 6Y2C (0.78-log_10_ cfu/mL; Supplementary Figure S1). Importantly, the combination of linezolid (16 mg/L) and rifampicin (0.5 mg/L) showed a synergistic bactericidal effect compared to each drug alone regardless of the presence of *cfr* gene (Supplementary Figure S1).

For *cfr*-negative MRSA group, the 50% maximal killing effect occurred at an average linezolid concentration of 0.71 mg/L (Figure 1B) and this decreased 3.4-fold to 0.21 mg/L in the presence of sub-MIC levels of rifampicin (Supplementary Table S1; paired *t*-test, *P* < 0.01). Expression of the *cfr* gene increased linezolid concentrations required to achieve 50% maximal effect to 2.01 mg/L and were significantly higher than the *cfr*-negative test group (Table 2; *P* < 0.05). However, when combined with rifampicin, the concentration of linezolid required to achieve 50% maximal effect was only 0.34 mg/L for *cfr*-positive MRSA group (Figure 1C). In fact, this level was comparable to the concentration required to potentiate rifampicin for *cfr*-negative group (Table 2). Although the concentration of 0.34 mg/L linezolid was insufficient to inhibit growth of MRSA carrying the *cfr* gene, its combination with rifampicin provided a promising alternative to overcome MRSA infections irrespective of *cfr* expression.

**TABLE 2:** Calculated EC_50_ and Hill slope (*N*) values representing the antimicrobial potency of linezolid alone or with 0.5 × MIC rifampicin against *cfr*-positive and -negative MRSA strains.^a^

	**Hill plot PD parameters**					
**MRSA strains**	**Linezolid alone**			**Linezolid + 0.5 × MIC rifampicin**		
	**EC_50_^b^**	***N***	***R*^2^**	**EC_50_^c^**	***N***	***R*^2^**
*cfr*+	2.01 ± 0.53	3.95 ± 0.46	0.95 ± 0.02	0.34 ± 0.15	4.22 ± 0.54	0.96 ± 0.02
*cfr*−	0.71 ± 0.18	3.53 ± 0.87	0.94 ± 0.02	0.21 ± 0.05	3.66 ± 1.03	0.97 ± 0.02

**^*a*^*EC_50_, the linezolid concentration required to achieve 50% of maximal effect (E_*max*_); *N*, the Hill coefficient that described the slope of the dose-response curve. *^*b*^P* < 0.05 for EC_50_ of linezolid alone in *cfr*-positive vs. -negative strains. *^*c*^P* < 0.01 for EC_50_ of linezolid and rifampicin in combination vs. linezolid alone.*

### *In vitro* HDPs Susceptibility and Adherence to FN and Endothelial Cells

**FIGURE 2:**
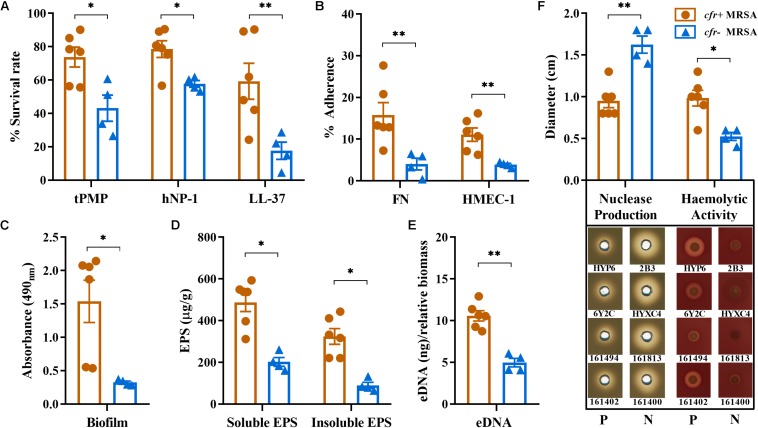
Relevant phenotype differences between *cfr*-positive and -negative MRSA strain. **(A)** *In vitro* susceptibilities to tPMP, hNP-1, and LL-37; **(B)** Adherence to immobilized FN and HMEC-1. **(C)** Biofilm formation by the study MRSA isolates based on absorbance values (OD_490__nm_). **(D,E)** Water-soluble and insoluble EPS and eDNA present in static biofilms. **(F)** Nuclease production and hemolytic activity of the study MRSA isolates on DNase agar and sheep blood agar plates, respectively. Diameters of zones clearance (cm) are indicated. Each dot represents one strain with three biological replicates. All data are presented as means ± SD. P, *cfr*-positive MRSA; N, *cfr*-negative MRSA (^∗^*P* < 0.01; ^∗∗^*P* < 0.005).

As a group, the *cfr*-positive MRSA exhibited significantly higher survival rates after exposure to 2 mg/L tPMP (73.8%) or 5 mg/L hNP-1 (78.5%) compared with the *cfr*-negative strain group (43.1 and 57.7%, respectively; *P* < 0.01). Similarly, a markedly reduced LL-37 killing was observed in *cfr*-positive vs. *cfr*-negative MRSA group (*P* < 0.005; Figure 2A). The *cfr*-positive MRSA demonstrated significantly higher binding rates to FN (14.8%) compared with the *cfr*-negative MRSA (4.02%; *P* < 0.005), despite a relatively low adherence to FN observed with the *cfr*-positive strain N4-2. Consistent with FN binding profiles, *cfr*-positive MRSA strain group bound substantially better to human endothelial cells than *cfr*-negative group (11.30 vs. 3.87%, *P* < 0.005; Figure 2B).

### Biofilm Formation, EPS and eDNA Determinations and Nuclease Productions

We compared *cfr*-positive and -negative MRSA strain groups with respect to *in vitro* biofilm capacity and composition. Overall, the *cfr*-positive MRSA group had a greater ability to form biofilms compared with the *cfr*-negative group (OD_490__nm_ 1.51 vs. 0.33, *P* < 0.01; Figure 2C). The production of the water-soluble EPS ranged from 161 to 593 μg/g, with the average production in *cfr*-positive group being considerably higher than that in *cfr*-negative group (487 μg/g vs. 202 μg/g, *P* < 0.01). A similar pattern was observed for the water-insoluble EPS between *cfr*-positive and -negative strain groups (*P* < 0.01; Figure 2D). Of note, the 24 h old MRSA biofilms showed increased production of the water-soluble EPS compared to water-insoluble EPS in both groups. In addition, the average amount of eDNA present in the *cfr*-positive MRSA biofilms (10.6 ± 1.37 ng) was 2.1-fold greater than that present in the *cfr*-negative MRSA biofilms (4.96 ± 0.88 ng), a statistically significant difference (*P* < 0.005; Figure 2E). These results were further corroborated by a significantly lower level of nuclease production in the *cfr*-positive MRSA group (*P* < 0.005; Figure 2F). In addition, except for strain 161494, the *cfr*-positive MRSA strain group possessed more α-hemolysin activity, compared with weak or non-detectable α-hemolysin production in *cfr*-negative MRSA group (Figure 2F).

### *In vivo* Responsiveness to Linezolid and Rifampicin Alone and in Combination

**FIGURE 3:**
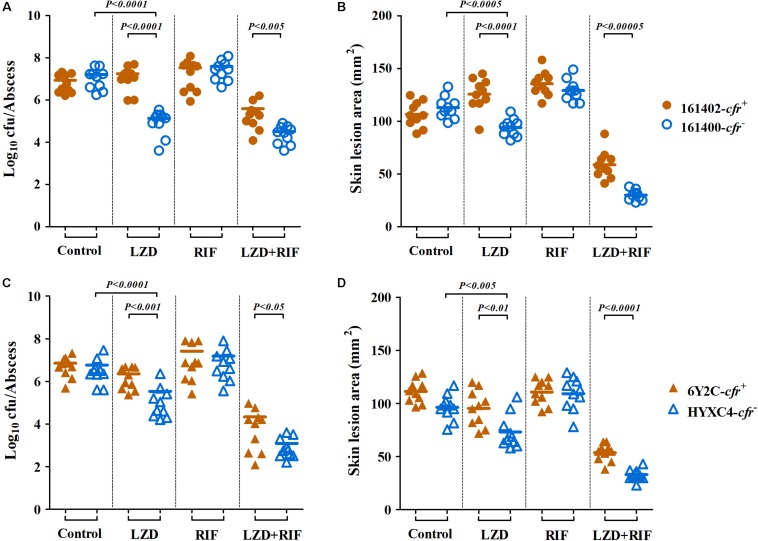
Therapeutic efficacy of linezolid (LZD; 100 mg/kg twice daily) or rifampicin (RIF; 5 mg/kg twice daily) alone and in combination in a murine SSSI model due to *cfr*-positive (161402 and 6Y2C) and -negative (161400 and HYXC4) MRSA strains. Each dot represents MRSA density in each skin abscess **(A,C)** or the area of each skin lesion **(B,D)**, and the horizontal lines represent the means of observations from groups of five mice (two abscesses per mouse).

In the murine SSSI model, MRSA densities in skin abscesses and the areas of skin lesions in mice infected with *cfr*-negative MRSA isolates 161400 and HYXC4 were significantly reduced after 5 days of linezolid monotherapy as compared to their untreated controls (*P* < 0.005, Figure 3). In contrast, for *cfr*-positive MRSA isolates 161402 and 6Y2C, the mice did not respond to linezolid monotherapy and residual abscess-tissue MRSA densities and the areas of skin lesions similar to those in their respective controls (Figure 3). Of note, combination therapy with linezolid and rifampicin resulted in 1.5–3.6 log_10_ cfu/abscess reductions in MRSA densities vs. linezolid monotherapy against both *cfr*-positive and -negative MRSA infections (Figures 3A,C). This result occurred despite the lower MRSA densities in skin abscesses observed in *cfr*-negative isolates (*P* < 0.05; Figure 3).

**FIGURE 4:**
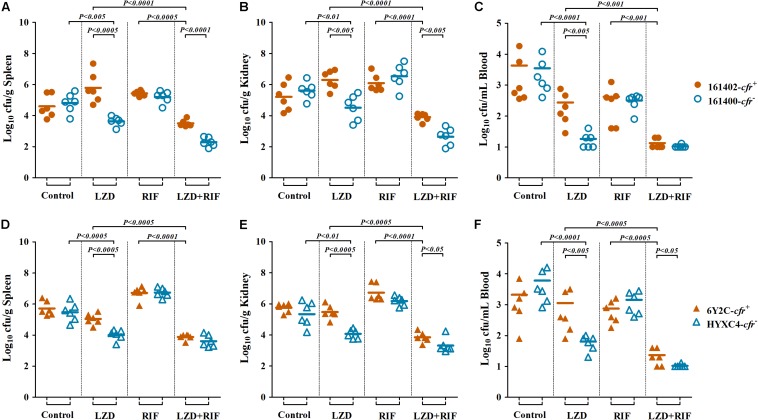
MRSA densities in spleen **(A,D)**, kidney **(B,E)**, and blood **(C,F)** in the murine bacteremia model due to *cfr*-positive (161402 and 6Y2C) and -negative (161400 and HYXC4) MRSA strains with linezolid or rifampicin mono- and combination therapies (LZD at 100 mg/kg, RIF at 5 mg/kg, orally twice a day for 3 days, starting at 24 h post-infection). Each dot represents one mouse, and the horizontal lines indicate the means of observations from groups of six mice.

In the bacteremia model, we found the similar results. Linezolid monotherapy resulted in uniform and highly significant reductions of MRSA densities in all the target tissues of mice infected with *cfr*-negative MRSA isolates 161400 and HYXC4. For instance, a ≥2.0 log_10_ cfu/mL reductions in blood density and ≥1.0 log_10_ cfu/g reductions in spleen and kidney densities were observed with linezolid monotherapy as compared to their respective control mice infected with *cfr*-negative MRSA (*P* < 0.01). However, bacteremia caused by *cfr*-positive MRSA isolates showed the opposite results: (i) a ≥1.0 log_10_ cfu/g increases in both spleen and kidney (161402, Figures 4A,B) or (ii) no response to linezolid monotherapy, with similar residual target-tissue MRSA densities compared with untreated controls (6Y2C; Figures 4D–F). Importantly, the combination of linezolid and rifampicin showed efficacies higher than each monotherapy in mice infected with both *cfr*-positive and -negative MRSA (*P* < 0.001; Figure 4).

## Discussion

*S. aureus* is a common opportunistic pathogen that causes a wide range of infections affecting skin and soft tissues as well as invasive infections that include bacteremia, endocarditis and pneumonia (Bassetti et al., 2014; Hassoun et al., 2017). In particular, bacteremia caused by MRSA is often associated with a high mortality rate, even with appropriate antibiotic treatments (Seidl et al., 2011a). Linezolid has been an important drug for therapy of MRSA infection. However, the emergence and rapid spread of horizontally transferable *cfr* determinant in MRSA has become a substantial concern (Witte and Cuny, 2011). The effective therapeutic alternatives were limited, especially for the vancomycin-intermediate and linezolid-resistant MRSA carrying *cfr* gene (Barber et al., 2016). Fortunately, unlike the chromosomally encoded resistance mechanisms, *cfr* has been shown to confer the low-level resistance to linezolid (Locke et al., 2014). Therefore, the combination therapy is an appealing option for retaining the clinical utility of linezolid.

Linezolid is a valuable alternative to glycopeptide antibiotics (e.g., vancomycin) and an oral formulation allows a rapid intravenous to oral switch (Dryden, 2011; Bassetti et al., 2014). As described in previous clinical studies, linezolid achieved significantly greater efficacy (e.g., higher cure rates) and earlier discharges from hospital than vancomycin treatment in patients with MRSA-complicated SSSI (Sharpe et al., 2005). This clinical outcome may be ascribed to a good penetration into skin and soft tissues with almost 100% oral bioavailability (Bassetti et al., 2014). Furthermore, linezolid treatments for MRSA bacteremia had roughly equivalent clinical and microbiological outcomes compared with vancomycin (Shorr et al., 2005). In our previous pneumonia model, addition of rifampicin to linezolid significantly decreased fAUC/MIC targets in both plasma and lung epithelial lining fluid (Zhou et al., 2018). Here, we extended the effectiveness of linezolid and rifampicin combination to the clinically relevant murine models of SSSI and bacteremia due to *cfr*-positive and -negative MRSA. More importantly, rifampicin decreases *S. aureus* FN binding that is a further advantage of the combination therapy (Rasigade et al., 2011). This is supported by other studies showing successful clinical outcomes with oral linezolid and rifampicin combination therapy in the management of recurrent and persistent MRSA bacteremia (Schwalm et al., 2004).

Indeed, we observed significant phenotype differences between *cfr*-positive and -negative MRSA that contribute to bacteremia and SSSI. The capability of *S. aureus* to circumvent clearance mediated by locally secreted HDPs is an important factor for its pathogenicity (Seidl et al., 2011a). In the current study, we demonstrated that *cfr*-positive MRSA isolates tended to be more resistant to key innate HDPs from neutrophils (hNP-1), platelets (tPMPs), and epithelial cells (LL-37), compared with the *cfr*-negative MRSA isolates. These findings suggested that *cfr*-positive MRSA might be more capable to survive in the bloodstream early in the course of skin infections. In particular, survival rates of >40% after 2 h of exposure to HDPs were positively correlated with the severity of endovascular infections and reduced responsiveness to antimicrobial therapy (Yeaman et al., 1992; Seidl et al., 2011a). Similarly, we observed a dramatic relationship between the reduced HDP killing *in vitro* and decreased efficacy of linezolid-based therapy in the murine bacteremia model.

Interestingly, *cfr*-positive MRSA group exhibited greater biofilm formation and higher EPS and eDNA productions compared with *cfr*-negative strain group. Biofilms enhance bacterial resistance to HDPs and antibiotics due to poor penetration past this barrier and this was the case for *cfr*-positive MRSA (Donlan and Costerton, 2002). Cathelicidin LL-37 can protect against MRSA-induced skin infections but *cfr*-positive MRSA were also able to resist the adverse effects of LL-37 exposure (Haisma et al., 2014). The alterations may contribute to the poor outcomes of linezolid therapy in mice infected with *cfr*-positive MRSA isolates in the SSSI model.

Invasive *S. aureus* must attach to extracellular matrix ligands or surface proteins on host cells to enable adhesion and internalization (Xiong et al., 2009). Therefore, the ability to bind FN is necessary for inducing *S. aureus* infections (e.g., bacteremia) (Seidl et al., 2011a). Consistent with this, increased FN binding of *cfr*-positive MRSA correlated with worse outcomes of linezolid-based mono- and combination therapies in the murine bacteremia model. This observation is also in line with previous reports that the development of a hyper-adhesive FN binding phenotype contributed to persistent MRSA bacteremia and infective endocarditis (Xiong et al., 2009, 2015). In addition, we found that *cfr*-positive MRSA isolates adhered better than *cfr*-negative isolates to human endothelial cells, and this may facilitate MRSA colonization and provide an advantage in the pathogenesis of invasive MRSA infections.

The exact mechanisms how the presence of *cfr* correlates with the phenotypic characteristics remain to be fully elucidated. In the recent past, it was assumed that the development of antibiotic resistance was linked to virulence and fitness costs (Beceiro et al., 2013). However, the acquisition of *cfr* in *S. aureus* has been associated with low fitness cost that potentially facilitates the growth rates, invasiveness and transmission capacity (LaMarre et al., 2011; Beceiro et al., 2013). In addition, in most of our study MRSA strains, *cfr* co-expressed with the *erm* gene (erythromycin resistance) that has a significant correlation with biofilm formations (Beceiro et al., 2013; Li et al., 2018). Previous studies in *Enterococcus* species exhibited that strong biofilm formation was more prevalent among linezolid-resistant compared with -sensitive isolates (Osman et al., 2020). *S. aureus* is a highly adaptable bacterium capable of dynamic changes in its virulence and resistance phenotypes when exposure to the host defenses or antibiotics (Abdelhady et al., 2015). Thus, additional unknown mechanisms likely contribute to the adaptive response phenotypes *in vitro* and linezolid-associated outcomes *in vivo*. Studies including comparative genomic and transcriptomic analysis are in progress in our laboratories to further determine other possible mechanisms.

Our investigation has several limitations. For example, we assessed a relatively small number of *cfr*-positive and -negative MRSA strains despite the different clonal types that raise the possibility of strain-dependent bias. In addition, some strains were isolated from animals and we only examined four representative MRSA strain sets in our animal models. Future studies should examine the phenotypic characteristics and usefulness of this combination in a larger population of strains from patients and in the clinical settings. Moreover, based on current findings, we do not know whether the relationship between the phenotypic profiles and the outcome of linezolid-based treatment is “*cfr* specific” or other unknown mechanisms. Although this is beyond the scope of the present study, future mechanism-based studies is warranted to better understand the precise factors responsible for this potential correlation.

Of note, the previous study reported that bacteriostatic-bactericidal antibiotic combinations could result in attenuation of bactericidal activity (Lobritz et al., 2015). However, our results showed the synergistic bactericidal effect for linezolid and rifampicin combination. This is supported by the previous observation that the combination of linezolid and rifampicin resulted in 3.1-log_10_ cfu/mL killing *in vitro* against staphylococcal biofilm (El Haj et al., 2018). Similarly, linezolid used in combination with rifampicin was more effective than their monotherapies, reducing the planktonic MRSA cells by >3.0 log_10_ cfu/mL in the cage fluids of foreign-body infections (Baldoni et al., 2009). In light of the divergent effects that observed between our results and previous study, future investigation is warranted to better understand the precise mechanism of this combination.

In summary, our results indicated that increased FN and endothelial cell adhesion, reduced susceptibility to HDPs and robust biofilm formation all contributed to linezolid treatment outcomes we found *in vivo*. Combination therapy with linezolid and rifampicin significantly enhanced therapeutic efficacy against experimental bacteremia and SSSI due to *cfr*-positive and -negative MRSA isolates.

## Data Availability Statement

The raw data supporting the conclusions of this article will be made available by the authors, without undue reservation, to any qualified researcher.

## Ethics Statement

This study was carried out in accordance with the recommendations of ethical guidelines of South China Agricultural University (SCAU). The protocol of *in vivo* studies and isolation procedures for animal-origin strains were approved by the SCAU Institutional Animal Ethics Committee (approval no. 2017B075). Individual written informed consent for the use of samples was obtained from all animal owners. Human-origin strains were kindly provided by the Third Affiliated Hospital of Sun Yat-sen University (Guangzhou, China), and isolation procedures were carried out in accordance with relevant guidelines with written informed consent from all subjects. The isolation and use protocols of human-origin strains were reviewed and approved by the Human Research Protection Office of SCAU Institutional Ethics Committee (approval no. 2017018).

## Author Contributions

Y-HL, YX, and Y-FZ designed the study. Y-FZ, LL, and M-TT carried out the experiments. Y-FZ, JS, and X-PL analyzed the data. Y-FZ wrote the manuscript. LL and YX revised the manuscript. All authors read and approved the final manuscript.

## Conflict of Interest

The authors declare that the research was conducted in the absence of any commercial or financial relationships that could be construed as a potential conflict of interest.
