# Supplementary material for: Linezolid and Rifampicin Combination to Combat cfr-Positive Multidrug-Resistant MRSA in Murine Models of Bacteremia and Skin and Skin Structure Infection
Source: Front Microbiol. 2020 Jan 14;10:3080. doi: 10.3389/fmicb.2019.03080 (PMC6971047; doi:10.3389/fmicb.2019.03080)
Supplement: Supplementary file 1 [file Data_Sheet_1.pdf]

Supplementary Table and Figure

Table S1. Calculated EC_{50} and Hill slope (N) values representing the antimicrobial potency of linezolid alone or with 0.5×MIC rifampicin against each *cfr*-positive and -negative MRSA strain. [a]

Organism	Hill plot PD parameters					
	Linezolid alone			Linezolid+0.5×MIC rifampicin		
	EC_{50} [b]	N	R^2	EC_{50} [c]	N	R^2
***cfr*-positive MRSA**						
161402	3.35	2.00	0.96	0.53	4.22	0.97
161494	3.22	6.26	0.98	0.52	4.49	0.99
N50	1.43	3.53	0.92	0.21	4.78	0.95
6Y2C	2.22	3.91	0.94	0.39	4.70	0.91
HYP6	0.94	4.57	0.98	0.21	3.93	0.96
N4-2	0.92	3.40	0.94	0.14	3.21	0.97
**Mean±SD**	**2.01±0.53**	**3.95±0.46**	**0.95±0.02**	**0.34±0.15**	**4.22±0.54**	**0.96±0.02**
***cfr*-negative MRSA**						
161400	0.61	2.46	0.97	0.21	2.92	0.94
161813	0.49	4.51	0.91	0.13	4.86	0.98
HYXC4	0.78	2.89	0.96	0.21	4.47	0.99
2B3	0.96	4.27	0.93	0.28	2.39	0.97
**Mean±SD**	**0.71±0.18**	**3.53±0.87**	**0.94±0.02**	**0.21±0.05**	**3.66±1.03**	**0.97±0.02**

[a] EC_{50}, the linezolid concentration required to achieve 50% of maximal effect (E_{max}); N, the Hill coefficient that described the slope of the dose-response curve. [b] $P<0.05$ for EC_{50} of linezolid alone in *cfr*-positive versus -negative strains. [c] $P<0.01$ for EC_{50} of linezolid and rifampicin in combination versus linezolid alone.

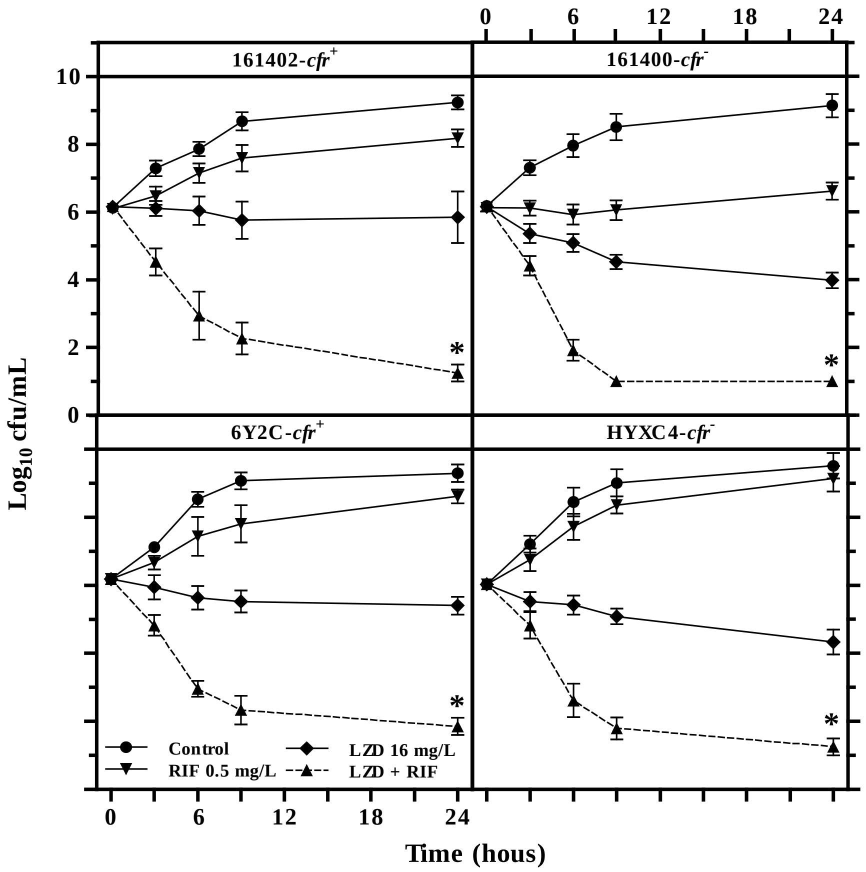

Figure S1. *In vitro* time-kill curves of linezolid (16 mg/L) and rifampicin (0.5 mg/L) alone and in combination against *cfr*-positive (161402 and 6Y2C) and -negative (161400 and HYXC4) MRSA strains. **P*<0.01, linezolid and rifampicin combination resulted in a ~3.0 log_{10}cfu reduction compared to each drug alone.
